# Atomically Dispersed Fe-N_4_ Modified with Precisely Located S for Highly Efficient Oxygen Reduction

**DOI:** 10.1007/s40820-020-00456-8

**Published:** 2020-05-26

**Authors:** Yin Jia, Xuya Xiong, Danni Wang, Xinxuan Duan, Kai Sun, Yajie Li, Lirong Zheng, Wenfeng Lin, Mingdong Dong, Guoxin Zhang, Wen Liu, Xiaoming Sun

**Affiliations:** 1 State Key Laboratory of Chemical Resource Engineering, Beijing Advanced Innovation Center for Soft Matter Science and Engineering, Beijing University of Chemical Technology, Beijing, 100029 People’s Republic of China; 2 Shandong University of Science and Technology, Electrical Engineering and Automation, Tsingtao, 266590 People’s Republic of China; 3 Department of Chemical Engineering, Loughborough University, Loughborough, Leicestershire LE11 3TU UK; 4 Interdisciplinary Nanoscience Center (INANO), Sino-Danish Center for Education and Research (SDC), Aarhus University, 8000 Aarhus C, Denmark; 5 Beijing Synchrotron Radiation Facility, Institute of High Energy Physics, Chinese Academy of Sciences, Beijing, 100049 People’s Republic of China

**Keywords:** Atomic dispersion, Iron–nitrogen moiety, Electronic structure, Sulfur doping, Oxygen reduction

## Abstract

Immobilizing metal atoms by multiple nitrogen atoms has triggered exceptional catalytic activity toward many critical electrochemical reactions due to their merits of highly unsaturated coordination and strong metal-substrate interaction. Herein, atomically dispersed Fe-NC material with precise sulfur modification to Fe periphery (termed as Fe-NSC) was synthesized, X-ray absorption near edge structure analysis confirmed the central Fe atom being stabilized in a specific configuration of Fe(N_3_)(N–C–S). By enabling precisely localized S doping, the electronic structure of Fe-N_4_ moiety could be mediated, leading to the beneficial adjustment of absorption/desorption properties of reactant/intermediate on Fe center. Density functional theory simulation suggested that more negative charge density would be localized over Fe-N_4_ moiety after S doping, allowing weakened binding capability to *OH intermediates and faster charge transfer from Fe center to O species. Electrochemical measurements revealed that the Fe-NSC sample exhibited significantly enhanced oxygen reduction reaction performance compared to the S-free Fe-NC material (termed as Fe-NC), showing an excellent onset potential of 1.09 V and half-wave potential of 0.92 V in 0.1 M KOH. Our work may enlighten relevant studies regarding to accessing improvement on the catalytic performance of atomically dispersed M-NC materials by managing precisely tuned local environments of M-N_x_ moiety.

**Electronic supplementary material:**

The online version of this article (10.1007/s40820-020-00456-8) contains supplementary material, which is available to authorized users.

## Highlights

- Precisely located S doping of atomic Fe-N_4_ in Fe(N_3_)(N–C–S) motif was realized.
- This S doping renders weakened *OH binding and faster charge transfer on Fe-N_4_.
- Fe-NSC showed excellent oxygen reduction reaction performance with onset potential ~ 1.09 V and half-wave potential ~ 0.92 V.

**Electronic supplementary material:**

The online version of this article (10.1007/s40820-020-00456-8) contains supplementary material, which is available to authorized users.

## Graphical abstract

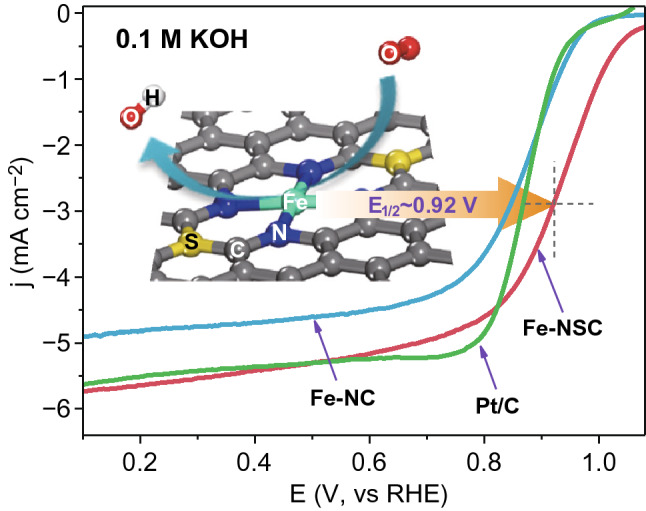

## Introduction

Atomically dispersed metal-nitrogen-carbon (M-NC) materials have received considerable attention due to excellent catalytic performance that boost sustainable and clean energy utilization technologies such as fuel cells [1–3], metal-air batteries [4–6], water electrolyzers [7–9], CO_2_/N_2_ fixation [10–13]. Their exceptional catalytic performance, as has been extensively studied, is firstly owing to the isolation presence of metal atom in high unsaturated coordination and high surface energy, secondly thanks to the strong beneficial interaction effects between atomic metal center and conductive substrate [14–17]. Besides of stabilizing metal centers via M-N_4_ moiety, the multiple N atoms surrounding metal center also act as bridges for communicating electrons in and out of metal centers and coordinators for responding the feedback from active substrate [18–21]. Therefore, it is of great importance and flexibility to tune the catalytic properties of central metal sites by simply manipulating the configuration and functionality of the coordinated N species.

Nowadays, most researches regarding atomically dispersed M-NC materials are being focused on the characterizations of active sites by using cutting-edge characterization techniques [22–25], and developments of new types of metal centers to fit more applicable fields [26–29], leaving large space for finely tuning the electronic properties of M-N_*x*_ moieties via local heteroatom doping. Currently, there have been some literatures reporting that the catalytic performance of atomically dispersed M-NC materials is highly related with rational design and optimization of the atomic structures of M-N_*x*_ moieties through adjusting their electronic properties, such as by breaking symmetry with *x* ≠ 4 [30–32], doping metal center or N periphery with heteroatoms [33–36]. However, it is still a big challenge to achieve optimal heterodoping through the trial-and-error introduction of heteroatom sources into starting materials. In order to achieve controllable dispersion of heteroatom dopants, distinct coupling and doping routes to the near adjacency of M-N_*x*_ moiety and clear understanding of heteroatom doping effects on the catalytic performance of M-N_*x*_ moiety are urgently called for.

Herein, the chemistry between formamide (N and C source) and thiourea (S source) was engaged to realize the local S doping to the atomically dispersed Fe–N_4_ moieties. The location of S atoms is confirmed by X-ray absorption near edge structure (XANES) analysis and Fe K-edge fitting in a configuration of Fe(N_3_)(N–C–S), which was inherited from the Shiff-base reaction pathway between amino groups on thiourea and carbonyl group on formamide [37]. Electrochemical measurements revealed that the S-doped atomically dispersed Fe-NC (shorted as Fe-NSC) material delivered excellent onset potential (~ 1.09 V) and half-wave potential (~ 0.92 V) for oxygen reduction reaction (ORR) in alkaline electrolyte. The significantly enhanced catalytic performance of Fe-NSC relative to S-free Fe-NC, in the first place, was ascribed to the isolated exposure of Fe atoms and in the second, was owing to the optimally regulated electronic structure by precisely doped S species. Density functional theory (DFT) computation suggested that more charge density would be accumulated around Fe(N_3_)(N–C–S) moiety relative to clean Fe-N_4_ moiety, which allowed weaker binding capability to *OH intermediates and faster charge transfer from Fe center to O species, thus leading to the enhancement of ORR performance.

## Experimental

### Materials

Formamide (FA, purity > 99%) was purchased from Xilong Chemical Co., Ltd. Zinc chloride (ZnCl_2_) and ferric chloride (FeCl_3_.4H_2_O) were purchased from Tianjin Fuchen Chemical Research Factory. Sulfuric acid (H_2_SO_4_, 95-98 wt %), and nitric acid (HNO_3_, 65 wt%) were purchased from Sinopharm Chemical Reagent Co., Ltd. N,N-dimethylformamide (DMF, purity > 99.5%) and thiourea (purity > 99.0%) were purchased from Aladdin reagent Co., Ltd. The commercial Pt/C catalyst (20 wt%, ~ 3 nm Pt nanoparticles on Vulcan XC-72 carbon support) was purchased from Shanghai MackLin biochemical technology co. LTD. Nafion/water or ethanol solution (~ 5 wt%) was purchased from Sigma-Aldrich Co., LTD. All chemicals were used as received without further purification.

### Preparations of Fe-NSC Material

The precursor for the preparation of Fe-NSC was synthesized via solvothermal method. In a typical synthesis [37], 0.005 mol L^−1^ FeCl_3_.4H_2_O (0.035 g), 0.01 mol L^−1^ thiourea (0.012 g), 0.1 mol L^−1^ ZnCl_2_ (0.408 g), and 30.0 mL formamide (FA) were mixed and sonicated for 30 min to get a homogeneous solution. Then, the mixture was transferred to autoclave and heated at 180 °C for 12 h. The as-formed brown precipitate was membrane-filtered, purified with deionized water 3 times and dried at 60 °C overnight. Then, the dried precursor was grinded in quartz mortar and heated to 900 °C for 2 h at a heating rate of 5 °C min^−1^ under Ar flow protection. The synthesis of control sample Fe-NC is like the Fe-NSC except for adding no thiourea.

### Characterizations

Transmission electron microscopy (TEM) images were recorded by a Hitachi-7700 operating at 100 kV. High-resolution transmission electron microscopy (HRTEM, operated at 200 kV) images were recorded using a JEOL 2100 high-resolution transmission electron microscope. High-angle annular dark field-scanning transmission electron microscope (HAADF-STEM) images and corresponding element mapping images were recorded on a JEOL JEM-ARM200F TEM/STEM with a spherical aberration corrector (operated at 300 kV). Powder X-ray diffraction (XRD) patterns were recorded from 5 to 80° at a scan rate of 10° min^−1^ using the Cu Kα 1.5406 Å radiation (MeasSrv F9XDZ42). Raman spectra were recorded on a LabRAM Aramis Raman spectrometer (HORIBA Jobin–Yvon, 500–3000 cm^−1^) with 532 nm line of Ar laser as excitation source. X-ray photoelectron spectroscopy (XPS) was recorded on a Thermo Electron ESCALAB250 XPS Spectrometer.

XAFS measurement and data analysis: XAFS spectra at the Fe K-edge were measured at the beamline 1W1B station of the Beijing Synchrotron Radiation Facility (BSRF), China. The Fe K-edge XAFS data of Fe-NSC were recorded in a fluorescence mode. Fe foil, FePc, and Fe_2_O_3_ were used as references in a transmission mode. The acquired EXAFS data were processed with the ATHENA module. The *k*^3^-weighted EXAFS spectra in the k-space ranging from 2 to 10.5 Å^−1^ were Fourier-transformed to real (*R*) space using a Hanning windows. The data fitting was done with Artemis software.

### Electrochemical Measurements

The working electrode was prepared by ultrasonically mixing 5.0 mg of catalyst sample with the mixture of 490 μL DMF and 10 μL 5% Nafion solution for 30 min to form homogeneous catalyst ink, and then, certain volume of the catalyst ink was cast-dropped onto polished glassy carbon rotating disk electrode (RDE, diameter ~ 5 mm) with a catalyst loading of 0.254 mg cm^−2^. The catalyst-modified RDE was submitted to electrochemical measurement of catalyzing ORR in both acid (0.1 mol L^−1^ HClO_4_) and alkaline electrolytes (0.1 mol L^−1^ KOH). Electrochemical measurements were carried out in a three-electrode setup on a Pine Modulated Speed Rotator with WaveDrive10 electrochemical workstation. Before ORR measurements, the O_2_ gas was bubbled into the electrolyte for at least 30 min to form O_2_ saturation. The cyclic voltammetry (CV) was collected at scan rate of 10 mV s^−1^. The polarization curves of ORR were collected at rotation speed of 400, 625, 900, 1225, 1600, and 2025 rpm with a scan rate of 5 mV s^−1^. The transferred electron number (n) and kinetic current density (*J*_K_) were calculated from the Koutecky–Levich equation [38]:

1 $$\frac{1}{J} = \frac{1}{{J_{\text{L}} }} + \frac{1}{{J_{\text{K}} }} = \frac{1}{{B\omega^{1/2} }} + \frac{1}{{J_{\text{K}} }}$$

2 $$B = 0.62{\text{nF}}C_{0} D_{0}^{{{\raise0.7ex\hbox{$2$} \!\mathord{\left/ {\vphantom {2 3}}\right.\kern-0pt} \!\lower0.7ex\hbox{$3$}}}} \upsilon^{{{\raise0.7ex\hbox{${ - 1}$} \!\mathord{\left/ {\vphantom {{ - 1} 6}}\right.\kern-0pt} \!\lower0.7ex\hbox{$6$}}}}$$

3 $$J_{\text{K}} = \frac{1}{{nKFC_{0} }}$$

where *J* is the measured current density; $J_{\text{K}}$ and $J_{\text{L}}$ are the kinetic and limiting current densities, respectively; *ω* is the angular velocity of the disk; n is the transferred electron number; F is the Faraday constant (96,485 C mol^−1^); $C_{0}$ is the bulk concentration of O_2_ (1.2 × 10^−6^ mol cm^−3^ in 0.1 mol L^−1^ KOH, and 1.26 × 10^−6^ mol cm^−3^ in 0.1 mol L^−1^ HClO_4_), *D*_0_ is the diffusion coefficient of O_2_ (1.9 × 10^−5^ cm^2^ s^−1^ in 0.1 mol L^−1^ KOH and 1.93 × 10^−5^ cm^2^ s^−1^ in 0.1 mol L^−1^ HClO_4_); and *υ* is the kinematic viscosity of the electrolyte (0.01 cm^2^ s^−1^ in 0.1 mol L^−1^ KOH and 1.009 × 10^−2^ cm^2^ s^−1^ in 0.1 mol L^−1^ HClO_4_). All the constants are data at 1 atm. and 25 °C.

### Calculation Models and Mechanism

Material Studio 5.5 software package was utilized to construct all the three Fe-NSC models based on a periodic 4  ×  4 graphene monolayer for the carbon framework. Subsequently, two carbon atoms were removed, followed by the substitution of four carbon atoms around the divacancy site with four nitrogen atoms to provide the anchoring site for iron atom. The carbon atoms adjacent to and opposite to the central Fe-N_4_ moiety were replaced by sulfur atoms, respectively, to stimulate the possible Fe-NSC structure, which further confirmed as Fe-NS_1_C, Fe-NS_2_C. C–S–C bonds were also considered and regarded as most likely existing state of S based on XPS analysis. Symmetrically, we constructed the existence situation of C–S–C bonds near the center of Fe-N_4_ moiety and defined it as the third possibility, Fe-NS_3_C. Oxygen reduction calculations were developed by the Viena Ab initio Simulation Package (VASP), by density functional theory (DFT + U, the description correlation for Fe is 3.5). Generalized Gradient Approximation (GGA) replaced the inner cores and the Perdew–Burke–Ernzerh (PBE) of functional were used to describe the exchange and correlation. The bulk lattice was optimized using the 3 × 3 × 3 Monkhorst–Pack type of K-point sampling with a period slab model for in the space of 16 Å vacuum. The cutoff energy was 400 eV, followed with an energy change convergence criterion of 1 × 10^−4^ eV. Spin polarization was also considered in all the calculations including the dipole correlation.

The stimulation of ORR process on active center of Fe-NSC was developed in the alkaline electrolyte. Thus, the four-electron transferred pathway, thus, the mechanism of oxygen reduction on single atom site could be normally treated as follows:

4 $${\text{O}}_{2} \left( g \right) + * + {\text{H}}_{2} {\text{O}}\left( l \right) + \, e^{-} \to {\text{OOH}}^{*} + {\text{OH}}^{ - }$$

5 $${\text{OOH}}^{*} + \, e^{-} \to {\text{O}}^{*} + {\text{OH}}^{ - }$$

6 $${\text{O}}^{*} + {\text{H}}_{2} {\text{O}}\left( l \right) + \, e^{-} \to {\text{OH}}^{*} + {\text{OH}}^{ - }$$

7 $${\text{OH}}^{*} + \, e^{-} \to * + {\text{OH}}^{ - }$$

The symbol of * stands for the active center which locate on the surface of catalytic bulk, and O*, OH*, and OOH* are adsorbed intermediates in the procedure of electrochemical reaction. Free Gibbs energy change (Δ*G*) from initial state to final state of oxygen reduction can be calculated as:

8 $$\Delta G = \, \Delta E + \, \Delta ZPE - \, T\Delta S + \, \Delta G_{U} + \, \Delta G_{pH}$$

where Δ*E* stands for the reaction energy change of adsorbed reactant and product molecules on the active sites of catalysts, by analyzing the DFT results. Δ*ZPE* and Δ*S* are the changes of zero-point energies and entropy due to the reaction, respectively. The Δ*G*_U_ shows the applied potential change relative to RHE, and Δ*G*_pH_ is the correction for H^+^ free energy.

## Results and Discussion

### Characterization of Fe-NSC

**Fig. 1:**
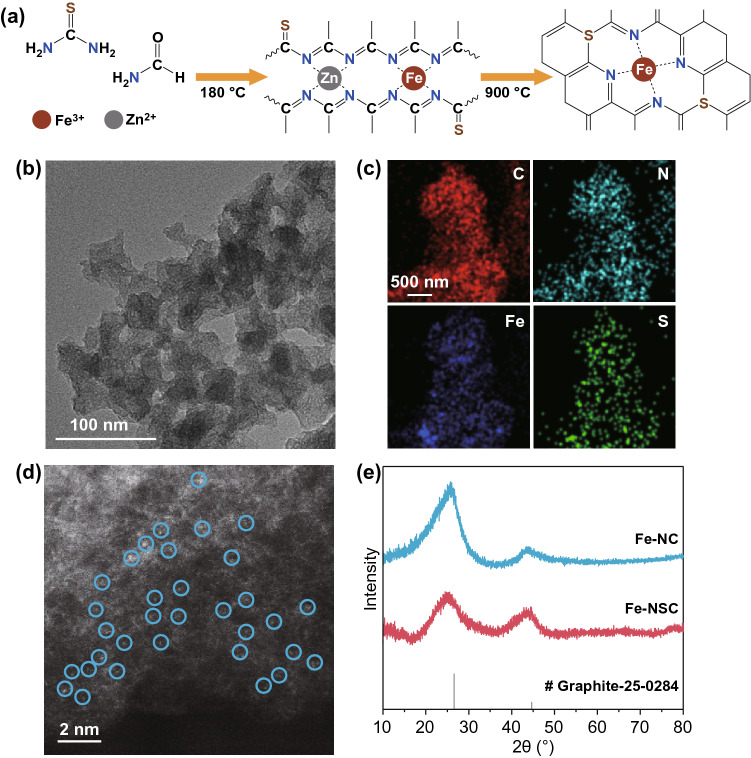
**a** Schematic illustration of the synthesis of Fe-NSC. **b** HRTEM and **c** element mapping images of Fe-NSC, **c** the uniform distribution of C (red), N (cyan), Fe (blue), and S (green) elements. **d** HAADF-STEM image of Fe-NSC, bright dots corresponds to the atomically dispersed Fe atoms. **e** XRD profiles of Fe-NC and Fe-NSC

As shown in Fig. 1a, the S-doped atomically dispersed Fe-NC materials, termed as Fe-NSC, were prepared via consecutive steps of solvothermal treatment and inert annealing. Formamide (FA) and thiourea molecules were utilized as C/N and S sources, respectively. During the solvothermal treatment, FA molecules crosslink via Shiff-base reaction to form multi-dentate polymeric ligands for anchoring ionic Fe^3+^ and Zn^2+^ [37]. In the meantime, thiourea molecules are able to incorporate to the neighbor of Fe-(N–C)_4_ moieties via Shiff-base reaction pathway between FA and thiourea. During the second step of high temperature annealing at 900 °C in N_2_ flow, carbothermic-reduced Zn substance vaporizes (boiling point of Zn is 906 °C), accompanying with the removal of unstable organic functionalities to reach high carbonization/graphitization degree [39]. Eventually, the targeted Fe-NSC material with atomically dispersed Fe sites, stable N_4_ surrounding, and S doping to the N_4_ moieties is obtained, as confirmed later in the section of XANES analysis. Therefore, our method based on FA chemistry may enable the adjustment on the charge density of atomic Fe center through N coordinators and surrounded S atoms.

The morphology of Fe-NSC was first probed using TEM and high-angle annular dark field-scanning transmission electron microscope (HAADF-STEM). The HRTEM image of Fe-NSC material in Fig. 1b shows even contrast, and no Fe aggregate is observed, implying the Fe components maintaining in cluster or possibly atomic dispersion. Meanwhile, the corresponding elemental mapping images confirm the uniform distribution of C, Fe, N, and S throughout the as-made Fe-NSC material (Fig. 1c). As indicated by the bright dots marked with circles (Fig. 1d), the HAADF-STEM image then confirms the Fe components in atomic dispersion in the N, S-codoped carbon matrix. Besides, no Fe aggregate and Fe components in atomic dispersion are seen in the control sample of Fe-NC obtained without thiourea (Fig. S1a, b), which mainly owes to the effects of Zn fencing and surrounding N immobilizing [40, 41].

The Fe-NSC and Fe-NC materials were further examined using XRD and Raman. As shown in Fig. 1e, the XRD profiles of Fe-NSC and Fe-NC exhibit two characteristic peaks at approximately 26.2° and 46.3°, which are indexing to the (002) and (100) plane of graphitic carbon, respectively. There is no diffraction additional peak observed that can be indexed to Fe-based compound or Fe substance for both Fe-NSC and Fe-NC, which agrees with HRTEM and STEM data, implying Fe components in high dispersion. Raman spectra (Fig. S1c) of Fe-NC and Fe-NSC show two evident bands locate at ~ 1352 and ~ 1591 cm^−1^ that, respectively, attribute to the D band (dispersive defect-induced vibration) and G band (the vibration of *sp*^2^-bonded carbon atoms) [42–44], indicating the high carbonization/graphitization feature of Fe-NSC and Fe-NC samples. The N_2_ adsorption/desorption measurement was employed to reveal the specific surface areas (SSA) and pore structures of Fe-NSC and Fe-NC. As indicated by the N_2_ adsorption and desorption (A/D) isotherms in Fig. S2a, both Fe-NSC and Fe-NC contain large amount of micropores and macropores, and small amount of mesopores that together build hierarchically porous structure. The BET SSA of Fe-NSC and Fe-NC are calculated to be 531.9 and 421.0 m^2^ g^−1^, respectively. The larger SSA of Fe-NSC implies that S doping could possibly induce more defects and structure distortion [44, 45]. Pore distribution curves are calculated using DFT method and showed in Fig. S2b, and it confirms the presence of considerable amounts of micropores and macropores presenting in both Fe-NSC and Fe-NC. Hierarchical porosity has been confirmed beneficial for facilitating electrolyte penetration and mass transportation in electrode materials [46–49].

**Fig. 2:**
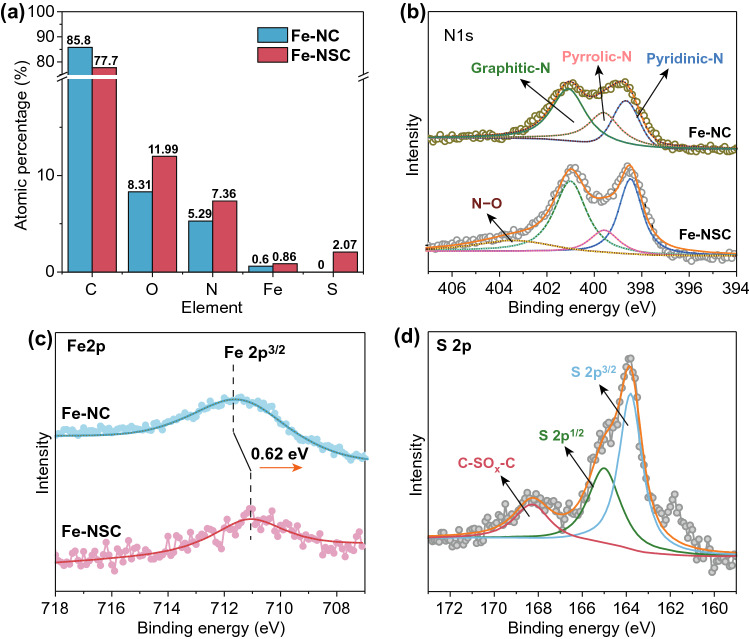
XPS analysis of Fe-NSC and Fe-NC. **a** Element contents, fine scans on **b** Fe 2*p* spectra, **c** N 1*s* spectra, and **d** S 2*p* spectra

X-ray photoelectron spectroscopy (XPS) was employed to analyze the composition of Fe-NSC and Fe-NC. XPS element surveys confirm the common presence of C, N, O, Fe elements in both Fe-NSC and Fe-NC (Fig. S3a), and Fig. 2a summarized the details of element contents in Fe-NSC and Fe-NC (see also in Table S1), in which Fe-NSC contains 77.71 at% C, 7.36 at% N, 0.86 at% Fe, and 2.07 at% S. In contrast, Fe-NC contains 85.80 at% C, 5.29 at% N, and 0.60 at% Fe. Detailed deconvolution of C 1*s*, N 1*s*, Fe 2*p*, and S 2*p* spectra was performed. Figure S3b reveals that more C–N and C–S connections are presenting in Fe-NSC than in Fe-NC, which is in accordance with element survey. With S introduction, more anchorable N atoms would stay in six-atom ringed pyridinic configuration (binding energy centered at ~ 398.5 eV) than in five-atom ringed pyrrolic form (binding energy centered at ~ 399.6 eV) (Fig. 2b) [50]. The XPS Fe 2*p* spectra in Fig. 2c show that both the Fe2*p*^3/2^ branches for Fe-NSC and Fe-NC spanning from 708 to 714 eV, suggesting the Fe components most likely presenting in their oxidative states. It is noteworthy that the band center of Fe-NSC shows approximately 0.5 eV blue-shifted compared to Fe-NC, meaning that the Fe atoms in Fe-NSC are in more reductive state, which is probably due to the close incorporation of S atom [51]. As shown in Fig. 2d, there are two main peaks located at ~ 164.0 and ~ 165.1 eV for the S species in Fe-NSC, which can be ascribed to the S 2*p*^3/2^ and S 2*p*^1/2^ branches of C-S-C moieties, and a minor peak centered at ~ 168.5 eV can be attributed to the sulfate species (–C–SO_*x*_–C, *x* = 1 or 2) [52–54].

**Fig. 3:**
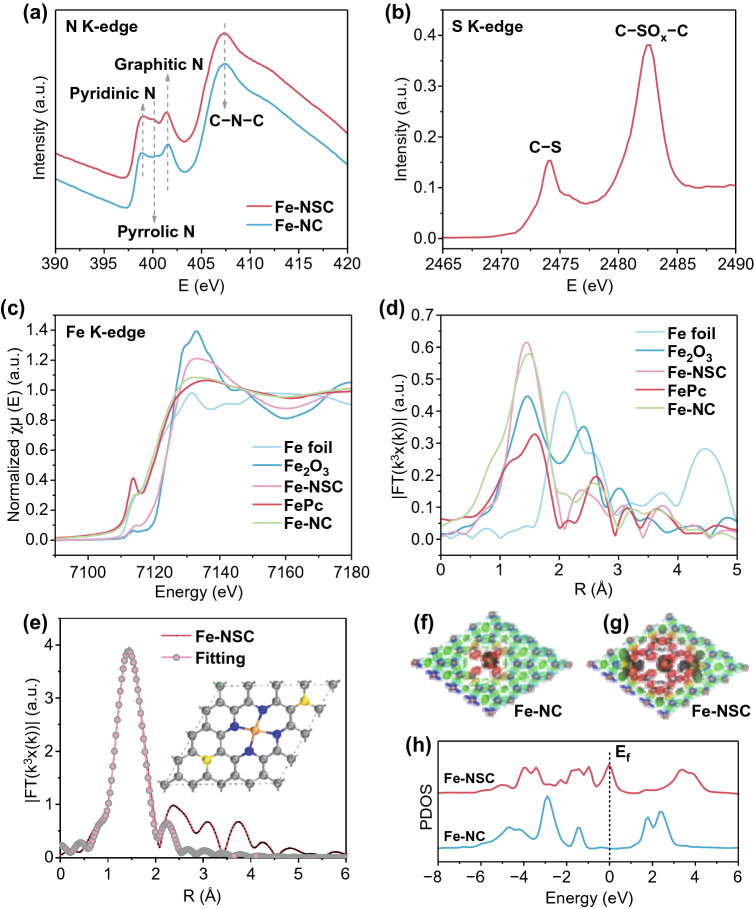
**a** N K-edge for Fe-NSC and Fe-NC, **b** S K-edge for Fe-NSC, and **c** experimental K-edge XANES Fe spectra of Fe-NSC, Fe-NC, and reference samples (Fe foil, FePc, and Fe_2_O_3_). **d** Fourier-transformed (FT) k^3^-weighted χ(k)-function of the EXAFS spectra for Fe K-edge of Fe-NSC, Fe-NC, and reference samples. **e** Corresponding Fe K-edge EXAFS fitting curves of Fe-NSC in R space, inset shows the proposed structure of Fe-N4 with local S doping. Top view of the charge densities of **f** Fe-NC (left) and **g** Fe-NSC (right), red area represents electron accumulation. **h** Partial density of states (DOS) for Fe d band in Fe-NSC and Fe-NC

The K-edge EXAFS of Fe-NSC and Fe-NC materials were measured to obtain deep insight into the local structures of active sites [55]. The N K-edge XANES spectra (Fig. 3a) reveal that the π* resonances at ~ 398, ~ 400.1, and 401.6 eV can be ascribed to the ringed structure consisting of pyridinic N, pyrrolic N, and quaternary N, respectively. The band at ~ 407.4 eV can be attributed to the σ* of C–N–C or C–N bonding [56, 57]. The Fe-NSC shows more evident π* resonance band at 400.1 eV, suggesting higher pyrrolic N content, which is in good agreement with XPS analysis. The S K-edge XANES (Fig. 3b) presents two typical peaks at 2474.2 and 2482.6 eV that can be indexed to C–S–C and C–SO_*x*_–C bonding, confirming the achievable S doping in Fe-NSC. The Fe K-edge XANES spectrum of Fe-NSC indicates its energy absorption threshold locating to the right of Fe-NC, Fe foil, and FePc, and closer to that of Fe_2_O_3_, implying the more positively charged Fe^δ+^ (2 < *δ* < 3) in Fe-NSC than in Fe-NC (Fig. 3c) [58]. FT-EXAFS spectrum (Fig. 3d) of Fe-NSC at Fe K-edge exhibits only one major peak at ~ 1.45 Å and no Fe–Fe bonding at ~ 2.09 Å, confirming the scattering Fe-N path. EXAFS fitting was performed to further manifest the coordination structure of Fe-N paths (Figs. 3e, S4 and Table S2) [57]. The fitting result shows that each Fe atom is coordinated by average four N atoms, giving the estimated structure of Fe-N_4_ with S doping at the S–C–N–Fe, as shown in the inset of Fig. 3e.

DFT study was conducted to identify the beneficial effect of neighboring S on Fe-N_4_ moiety. Firstly, the charge distribution and partial density of states (PDOS) were established by using a model with Fe-N_4_ sites embedded in N, S-co-doped carbon substrate to mimic the active sites of S-neighbored Fe-N_4_ moiety in Fe-NSC, as shown in Fig. 3f, g. Compared to that of Fe-NC, the charge distribution pattern of Fe-NSC shows strong electron accumulation (marked by red area) around Fe center. The extended electron accumulation around Fe-N_4_ moiety is attributed to neighboring S regulation, leading to more electron density partially accumulated on Fe centers [51]. The denser charge density of Fe-N_4_ moiety promoted by S regulation may help to accelerate charge transfer during ORR processes [59, 60]. The PDOS of Fe atoms in Fe-NSC and Fe-NC indicate that the gap of Fe d band in Fe-NSC structure is much narrower than that in Fe-NC (Fig. 3h), meaning S doping in Fe-NC also contributing the improvement on conductivity. Moreover, the highly localized d band center of Fe in Fe-NSC are up-shifted with reduced intensity, which leads to weaker binding capability to reactant O_2_ molecules and better electron transferring [59]. These above DFT simulation results strongly suggest that the electronic structure of Fe-N_4_ moieties has been efficiently regulated by neighboring S [61, 62].

### ORR Performance of Fe-NSC

**Fig. 4:**
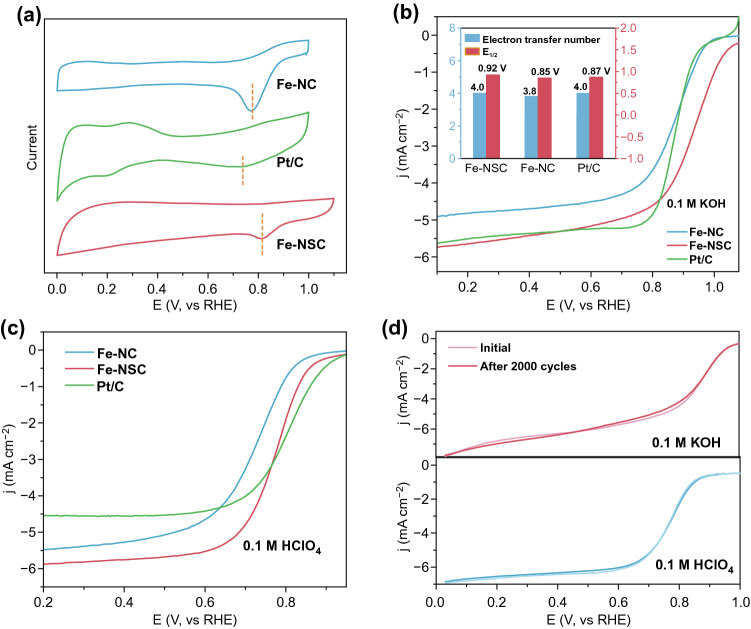
Electrochemical measurements on Fe-NSC, Fe-NC, and Pt/C. **a** CV profiles with scan rate of 100 mV s^−1^. LSV curves in O_2_-saturated **b** 0.1 M KOH and **c** 0.1 M HClO_4_ with a sweep rate of 5.0 mV s^−1^ and 1600 rpm. **d** Stability test of Fe-NSC in 0.1 M KOH (top) and 0.1 M HClO_4_ (bottom) for 2000 cycles (~ 12 h)

The as-obtained Fe-NSC, Fe-NC, and 20 wt % Pt/C (Pt/C) were submitted to ORR measurements. Their catalytic activity was first investigated by using cyclic voltammetry (CV) and linear sweep voltammetry (LSV) measurements in O_2_-saturated 0.1 mol L^−1^ KOH with the same catalyst loading of 0.254 mg cm^−2^. It is notable in the CV profiles (Fig. 4a) that Fe-NSC exhibits a remarkably high peak-current potential of 0.82 V at scan rate of 100 mV s^−1^, while the Fe-NC and Pt/C show lower peak-current potentials of 0.78 and 0.74 V, respectively. As examined by LSV, the onset potential of Fe-NSC is ~ 1.09 V, which largely surpasses those of Pt/C and Fe-NC for over 90 and 80 mV, respectively (Fig. 4b), which agrees with their corresponding CV curves. The achieved high onset potential of 1.09 V for ORR in alkaline is among the best values ever reported (Table S3). Meanwhile, the Fe-NSC catalyzed ORR shows an exceptional half-wave potential (*E*_1/2_) of 0.92 V in 0.1 mol L^−1^ KOH (inset of Fig. 4b), which is 50 and 70 mV positive compared to those of Pt/C (~ 0.87 V) and Fe-NC (~ 0.85 V). The electron transfer number (*n*) of Fe-NSC and Fe-NC was measured using LSV curves obtained at different rotation speeds with a scan rate of 5 mV s^−1^ (Fig. S5a, c). As displayed in Fig. S5b, the linearity of the Koutecky–Levich (K-L) plots of Fe-NSC indicates the first-order reaction kinetics and the *n* was calculated to be ~ 4.0 at the potential range of 0.2–0.7 V, which is slightly higher than Fe-NC (~ 3.8) (inset of Fig. 4b). Tafel plots were organized and displayed in Fig. S5e to further investigate the ORR kinetics [63]. The Fe-NSC, Fe-NC, and Pt/C, respectively, reach slopes of 102.8, 103.4, and 106.8 mV dec^−1^, suggesting the process of O_2_ to OOH* being determinant at such potential range [49]. The stability toward methanol crossover was investigated by using chronoamperometric technique [64]. A slight current oscillation is observed for Fe-NSC, leading to over 94.0% current retention, indicating good capability to tolerate methanol (Fig. S5f). By contrast, Pt/C exhibits sharp current drop to 38.8% at the moment of methanol injection and is not able to recover after the next 10 min. Electrochemical surface area (ECSA) of both samples was calculated based on their electrochemical double-layer capacitances (EDLCs). As shown in Fig. S6a–c, the Fe-NSC sample possesses a little bit larger ECSA of 8.6 mF cm^−2^ compared to the Fe-NC (~ 7.4 mF cm^−2^). We then normalized the specific kinetic current density of ORR using the ECSA, as displayed in Fig. S6d. This bar chart comparison shows that the kinetic current densities of Fe-NSC at three given potentials (0.60, 0.65, and 0.70 V) are higher than those of Fe-NC, further confirming the beneficial effect of S introduction to the adjacence of Fe-N_4_ moiety.

The ORR performance of Fe-NSC, Fe-NC and Pt/C in acidic electrolyte of 0.1 M HClO_4_ was also studied. Figure S7a shows their CV curves in acid electrolyte, manifesting the very close peak potential of ~ 0.67 V at peak current for Fe-NSC and Pt/C. The Fe-NSC exhibits an estimated *E*_1/2_ of 0.78 V, approaching to that of Pt/C (~ 0.80 V), as compared in Fig. 4c, which is 70-mV positive than that of S-free Fe-NC. Meanwhile, the Fe-NSC shows much larger limiting current density relative to that of Pt/C. Figure S7c shows the *n* of Fe-NSC that is calculated from LSV curves in Fig. S7b to be ~ 3.9, implying a near direct 4-electron pathway [65]. Figure S7d reveals that the lowest Tafel slope of 92.6 mV dec^−1^ is achieved by the Fe-NSC over Pt/C (~ 102.9 mV dec^−1^) and Fe-NC (~ 100.2 mV dec^−1^). In addition, the long-term cycling stability in both alkaline and acidic electrolytes was investigated by using chronoamperometric technique. As shown in Fig. 4d, very limited activity decays were observed in both alkaline and acid electrolytes for non-stop 12-h working, further supporting that Fe-NSC material is reasonably good ORR catalyst that can be potentially used for proton exchange membrane fuel cell^3^. Furthermore, HRTEM images of Fe-NSC catalyst after 12-h long working were collected and showed in Fig. S8, and no metal aggregates are seen which confirm the Fe-N_4_ sites are well-reserved in their original atomic dispersion after durability tests.

### DFT Calculations

**Fig. 5:**
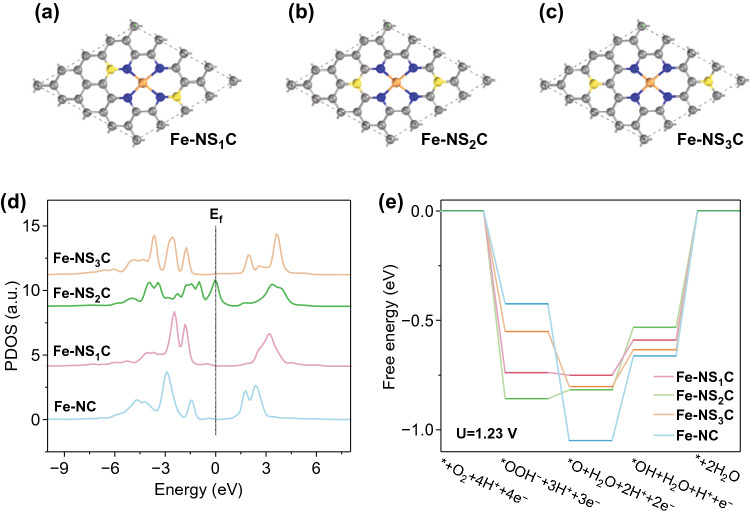
Top view of atomic structure of Fe-NSC with different S positions: **a** Fe-NS_1_C, **b** Fe-NS_2_C, and **c** Fe-NS_3_C. **d** Partial density of states (PDOS) of Fe d band in Fe-NSC with S doping at different local positions, with comparison to that of Fe-NC. **e** Free-energy diagram of ORR occurring on Fe-NSC with S doping at different local positions, obtained from DFT calculation

We simulated another two possible locations of S doping to the periphery of Fe-N_4_ moiety, and DFT calculations were employed to investigate the adsorption free energy of ORR steps, as well as to evaluate the difference of electronic structures with different S locations. Figure 5a–c displays three S location to Fe-N_4_ moiety, they are nominated as Fe-NS_1_C, Fe-NS_2_C, and Fe-NS_3_C according to their positions, and the subscript of S stands for the number sequence next to N-Fe. The projected PDOS of Fe atoms in Fe-NS_1_C, Fe-NS_2_C, Fe-NS_3_C, and Fe-N_4_C moieties are compared in Fig. 5d. The PDOS study on Fe atom in Fe-NS_*x*_-C suggested that the d band gap of Fe in Fe-NS_2_-C was much narrower than the Fe atoms in other Fe-NS_*x*_-C, which can be interpreted into the improved local electronic conductivity in Fe-N_4_ moiety by adjacent S doping. Figure S9 shows the geometry of each intermediates on Fe site in Fe-NS_3_C moiety during oxygen reduction catalysis. The free-energy diagrams of ORR promoted by Fe-NS_1_C, Fe-NS_2_C, Fe-NS_3_C, and Fe-NC are displayed in Fig. 5e. All the free-energy calculations were performed at U = 1.23 V for four-electron transfer reactions for oxygen reduction. In the first step of *O_2_ to *OOH, all the Fe-NS_*x*_C (x = 1, 2, or 3) show much larger energy release than Fe-N_4_ does, confirming their weaker binding capability to O_2_ molecule. The last step of ORR (*OH reduction) is revealed to be the most sluggish endothermic process and thus can be confirmed to be one rate-determining step (RDS) for both Fe-NSC and Fe-NC catalysts [66]. The overpotential of ORR promoted by Fe-NS_2_C is calculated to be 0.53 V, which 0.13 V lower than that by Fe-NC, and this improvement is likely contributed by S doping which reduced the adsorption capacity of *OH intermediate, thus facilitating the dynamic progress of ORR [36].

## Conclusions

In summary, we have developed a reliable strategy to boost the ORR performance of atomically dispersed Fe-N_4_ active sites by precise S local doping. The Fe-NSC material has been thoroughly characterized by XRD, XPS, HAADF-STEM, and XANES techniques, confirming the atomically dispersion of Fe component in S-neighbored Fe-N_4_ structure of Fe(N_3_)(N–C–S). The significant enhancement of ORR activity of Fe-NSC was confirmed by electrochemical measurements, exhibiting a remarkably high onset potential of 1.09 V and a *E*_1/2_ of 0.92 V in 0.1 M KOH, which were 90 and 50 mV higher than that of Pt/C, respectively. Meanwhile, the Fe-NSC showed highly comparable acidic ORR performance relative to Pt/C, delivering a 0.78 V *E*_1/2_ (only 20 mV less than that of Pt/C). The drastically improved ORR activity, as proved by DFT simulations, can be credited to the S doping-induced charge enrichment in N and Fe for optimal O_2_ binding and fast electron transferring. Our work, beside of rendering superior ORR catalyst in both alkaline and acid electrolytes, provided a feasible strategy of regulating the local electronic structure of M-N_4_ moiety by controllable doping with heteroatoms to improve catalytic performance.

## Electronic supplementary material

Below is the link to the electronic supplementary material.

Supplementary material 1 (PDF 476 kb)
