# Supplementary material for: Atomically Dispersed Fe-N4 Modified with Precisely Located S for Highly Efficient Oxygen Reduction
Source: Nanomicro Lett. 2020 May 26;12:116. doi: 10.1007/s40820-020-00456-8 (PMC7770948; doi:10.1007/s40820-020-00456-8)
Supplement: Supplementary file 1 — Supplementary material 1 (PDF 476 kb) [file 40820_2020_456_MOESM1_ESM.pdf]

Supporting information for

Atomically Dispersed Fe-N_4 Modified with Precisely Located S for Highly Efficient Oxygen Reduction

Yin Jia[1,†], Xuya Xiong[4,†], Danni Wang[2], Xinxuan Duan[1], Kai Sun[1,3], Yajie Li[1], Lirong Zheng[5], Wenfeng Lin[3], Mingdong Dong[4], Guoxin Zhang[2,*], Wen Liu[1,*], Xiaoming Sun[1,*]

[1]State Key Laboratory of Chemical Resource Engineering, Beijing Advanced Innovation Center for Soft Matter Science and Engineering, Beijing University of Chemical Technology, Beijing100029, People's Republic of China

[2]Shandong University of Science and Technology, Electrical Engineering and Automation, Tsingtao 266590, People's Republic of China

[3]Department of Chemical Engineering, Loughborough University, Loughborough, Leicestershire, LE11 3TU, UK

[4]Interdisciplinary Nanoscience Center (iNANO), Sino-Danish Center for Education and Research (SDC), Aarhus University, DK-8000 Aarhus C, Denmark

[5]Beijing Synchrotron Radiation Facility, Institute of High Energy Physics, Chinese Academy of Sciences, Beijing 100049, People's Republic of China

†Yin Jia and Xuya Xiong contributed equally to this work

*Corresponding authors. E-mail: zhanggx@sdust.edu.cn (G. Zhang), wenliu@mail.buct.edu.cn (W. Liu), sunxm@mail.buct.edu.cn (X. Sun)

Supplementary Figures

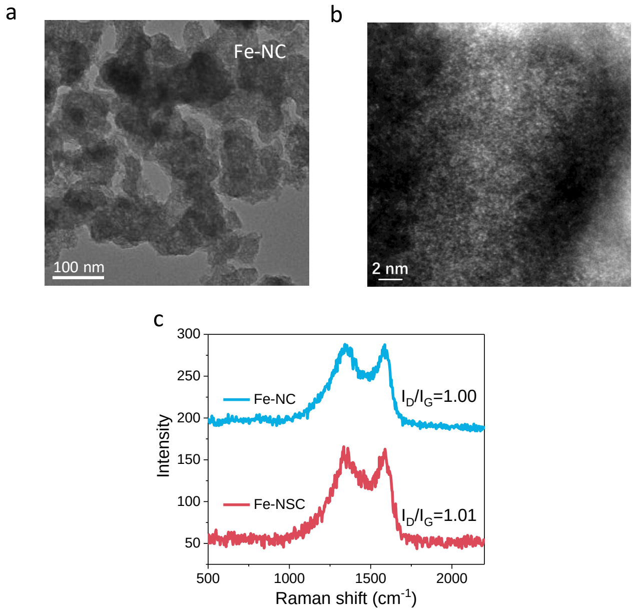

Fig. S1 (**a**) HRTEM, and (**b**) HAAD-STEM image of Fe-NC. (**c**) Raman spectra

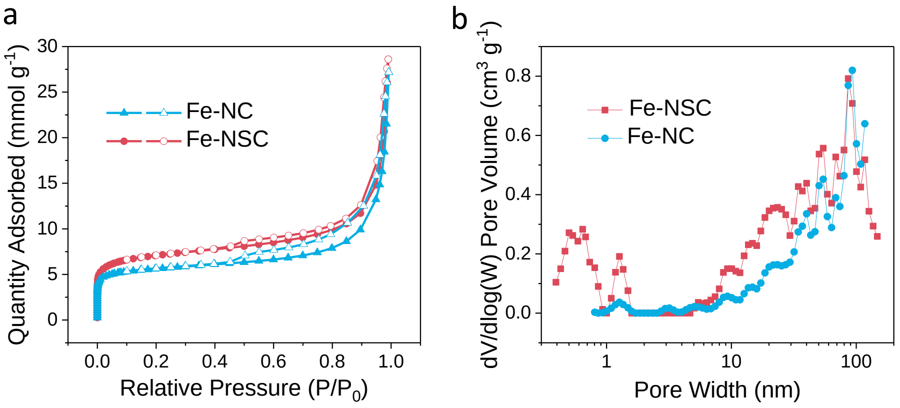

Fig. S2 (**a**) BET N_2 adsorption/desorption isotherms, and (**b**) pore distribution curves of Fe-NSC and Fe-NC

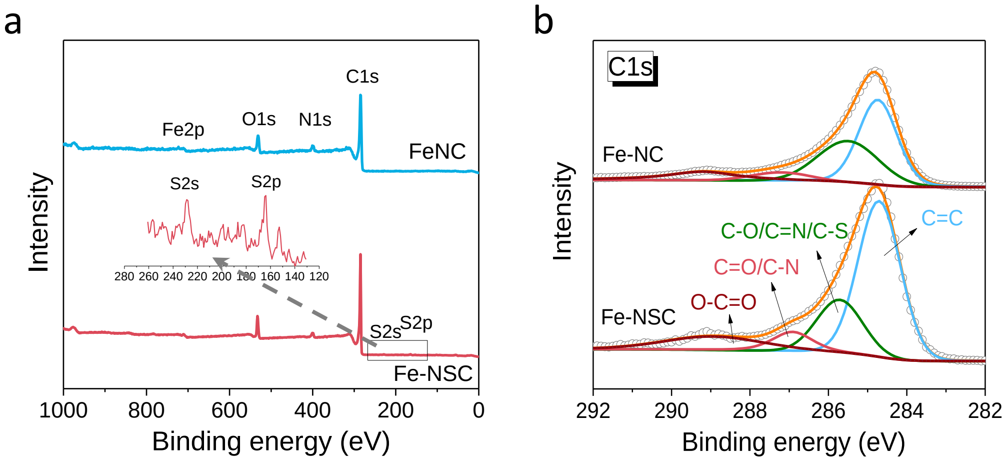

Fig. S3 (**a**) XPS surveys and (**b**) XPS C1s spectra fine scans of Fe-NSC and Fe-NC, inset of panel (**a**) exhibits the signal of S 2s and S 2p spectra in Fe-NSC

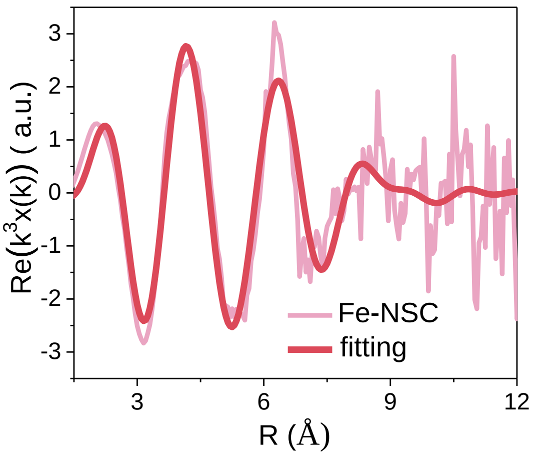

Fig. S4 Fe K-edge EXAFS fitting curves of Fe-NSC in k space

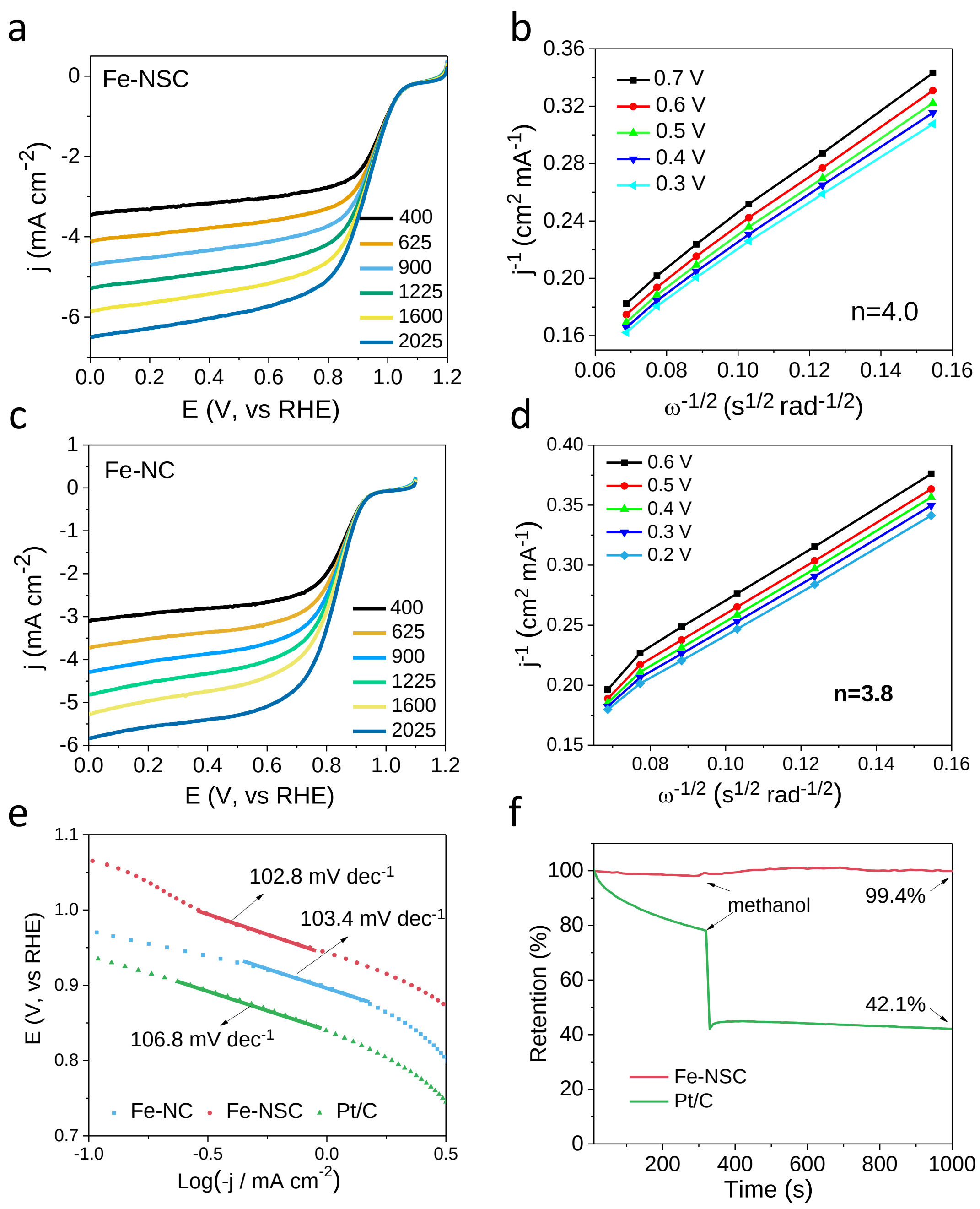

Fig. S5 ORR measurements in 0.1 mol L^{-1} KOH. (**a**) RDE voltammograms at different rotation speeds and (**b**) the corresponding Koutecky-Levich plots of Fe-NSC, (**c**) RDE voltammograms at different rotation speeds and (**d**) the corresponding Koutecky-Levich plots of Fe-NC. (**e**) Tafel plots of Fe-NSC, Fe-NC, and Pt/C. (**f**) Methanol crossover tests of Fe-NSC and Pt/C

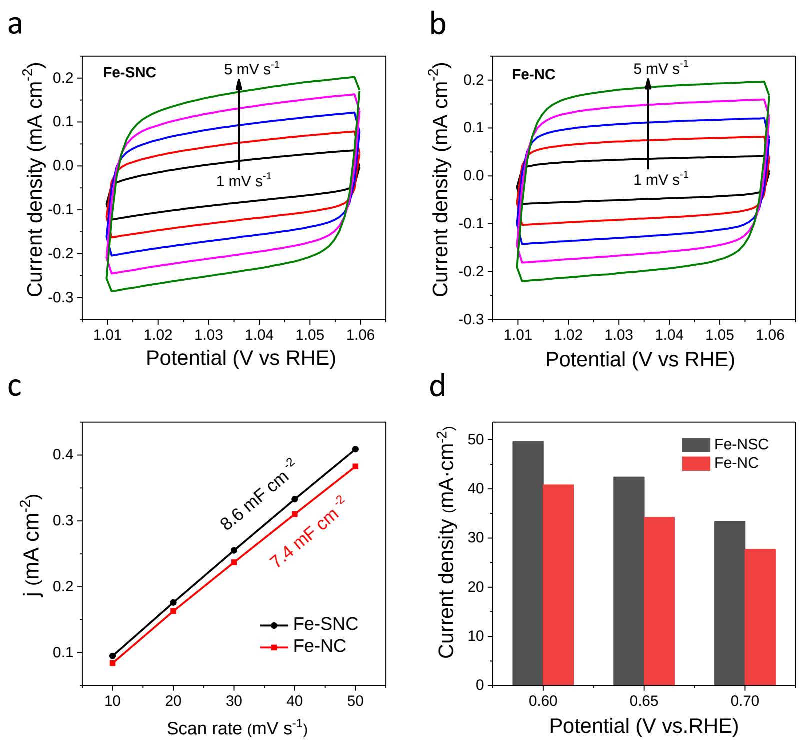

Fig. S6 CV measurements on (**a**) Fe-NSC and (**b**) Fe-NC samples, (**c**) ECSA of Fe-NSC and (**b**) Fe-NC samples. (**d**) Normalized ORR current density at different voltages using intercept method

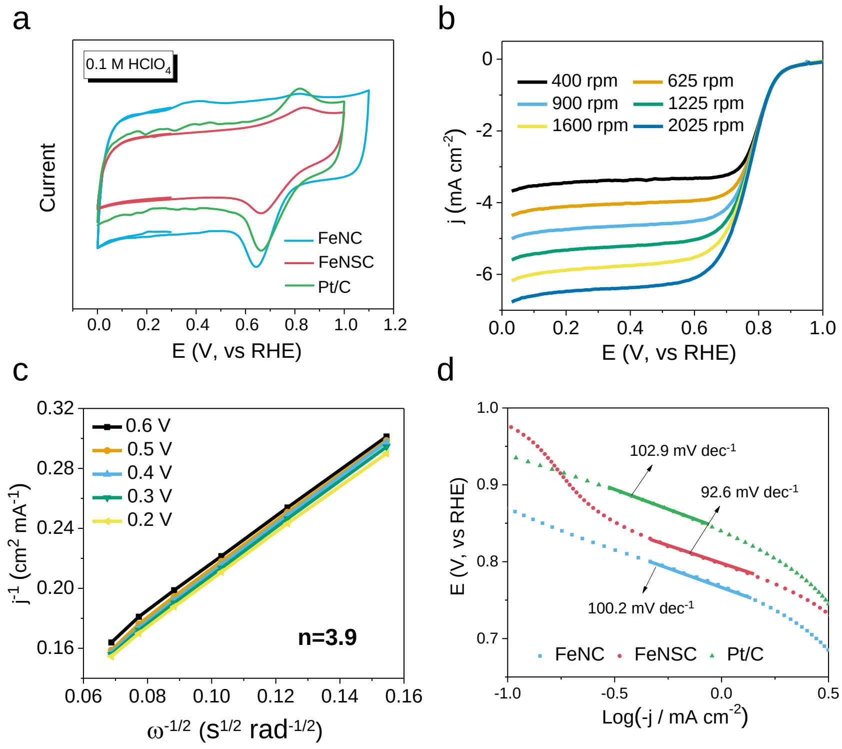

Fig. S7 ORR measurements in 0.1 mol L^{-1} $HClO_4$. (**a**) CV curves of Fe-NSC, Fe-NC, and Pt/C, (**b**) RDE voltammograms at different rotation speeds and (**c**) the corresponding Koutecky-Levich plots of Fe-NSC, (**d**) corresponding Tafel plots obtained from the RDE polarization curves

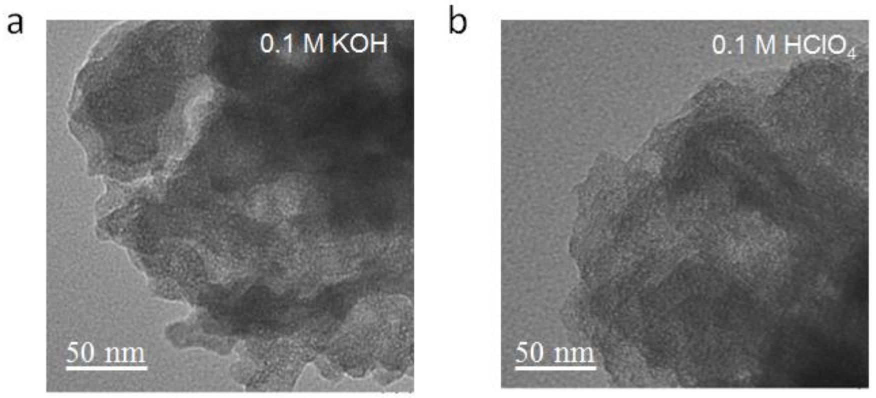

Fig. S8 TEM images of Fe-NSC after 2000 cycles in (**a**) 0.1 mol L^{-1} KOH and (**b**) 0.1 mol L^{-1} $HClO_4$

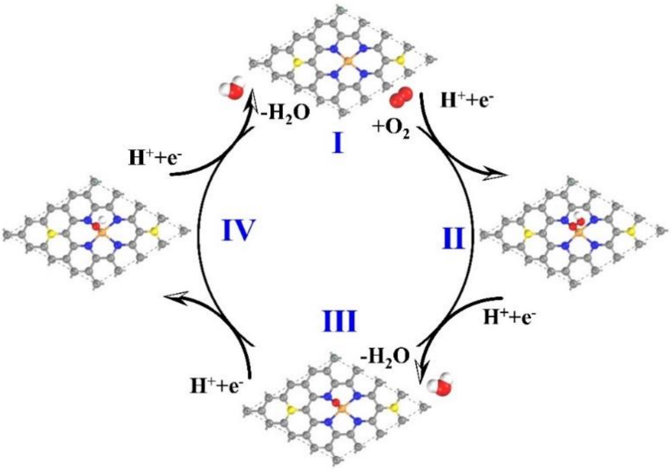

Fig. S9 Proposed reaction scheme of associative mechanism for ORR on Fe-NS_2C moiety

Supplementary Tables

Table S1 Element analysis of Fe-NC and Fe-NSC based on XPS characterizations

Sample	C (at%)	N (at%)	O (at%)	Fe (at%)	S (at%)
Fe-NC	85.80	5.29	8.31	0.60	-
Fe-NSC	77.71	7.36	11.99	0.86	2.07

Table S2 Structural parameters of Fe-NSC extracted from the EXAFS fitting

Sample	Scattering pair	CN	R(Å)	$\sigma^2(10^{-3}Å^2)$	R factor
Fe in Fe-NSC	Fe-N	4.4	2.07	0.09	0.005
Fe in Fe-NC	Fe-N	3.47	1.99	14.8	0.002

CN is the coordination number; R is interatomic distance (the bond length between central atoms and surrounding coordination atoms); σ^2 is Debye-Waller factor (a measure of thermal and static disorder in absorber-scatterer distances). R factor is used to value the goodness of the fitting.

* This value was fixed during EXAFS fitting, based on the known structure.

Error bounds that characterize the structural parameters obtained by EXAFS spectroscopy were estimated as N ± 20%; R ± 1%; σ^2 ± 20%; ΔE_0 ± 20%. Fe- (FT range: 2.0-10.5 $Å^{-1}$; fitting range: 0.5-3.0 Å)

Table S3 Comparison of ORR performance of non-precious metal-nitrogen-carbon materials in 0.1 M KOH

Sample	Potentials / V_{RHE}		Electron number	Tafel slope / mV dec^{-1}	References
	Onset	Half-wave			
Co SAs/N-C	1.0	0.881	~4.0	75	S1
Fe-N/C	0.923	0.809	4.15	-	S2
Fe_1/N,S-PC	1.0	0.904	3.95	84.5	S3
Fe/N/S-CNTs	0.987	0.887	~4.0	73	S4
cal-CoZIF-VXC72	0.93	0.84	~4.0	45	S5
ZnNx/BP	1.0	0.825	~4.0	-	S6
S,N-Fe/N/C-CNT	0.98	0.85	4.0	82	S7
Fe-ISA/SNC	1.0	0.896	3.91-3.98	44	S8
Fe-S,N-C	0.95	0.83	>3.94	59	S9
(Fe,Co)/CNT	1.15	0.954	4.0	-	S10
$FeCl_1N_4$/CNS	1.0	0.921	3.96-3.99	51	S11
Fe-SAs/NCP-HC	0.98	0.912	3.96-3.99	36	S12
Co–C_3N_4/CNT	0.9	0.85	4.0	68.4	S13
**Fe-NSC**	**1.09**	**0.92**	**4.0**	**102.8**	**This work**

Supplementary References

[S1] P. Yin, T. Yao, Y. Wu, L. Zheng, Y. Lin et al., Single cobalt atoms with precise N-coordination as superior oxygen reduction reaction catalysts. Angew. Chem. Int. Ed. **55**(36), 10800-10805 (2016). https://doi.org/10.1002/ange.201604802

[S2] L. Lin, Q. Zhu, A.W. Xu, Noble-metal-free Fe-N/C catalyst for highly efficient oxygen reduction reaction under both alkaline and acidic conditions. J. Am. Chem. Soc. **136**(31), 11027-33 (2014). https://doi.org/10.1021/ja504696r

[S3] K. Wu, X. Chen, S. Liu, Y. Pan, W.-C. Cheong et al., Porphyrin-like Fe-N_4 sites with sulfur adjustment on hierarchical porous carbon for different rate-determining steps in oxygen reduction reaction. Nano Research **11**(12), 6260-6269 (2018). https://doi.org/10.1007/s12274-018-2149-y

[S4] H. Jin, H. Zhou, W. Li, Z. Wang, J. Yang et al., In situ derived Fe/N/S-codoped carbon nanotubes from ZIF-8 crystals as efficient electrocatalysts for the oxygen reduction reaction and zinc–air batteries. J. Mater. Chem. A**.** **6**(41), 20093-20099 (2018). https://doi.org/10.1039/c8ta07849a

[S5] B. Ni, C. Ouyang, X.Xu, J. Zhuang, X. Wang, Modifying commercial carbon with trace amounts of ZIF to prepare derivatives with superior ORR activities. Adv. Mater. **29**(27) (2017). https://doi.org/10.1002/adma.201701354

[S6] P. Song, M. Luo, X. Liu, W. Xing, W. Xu, Z. Jiang, L. Gu, Zn single atom catalyst for highly efficient oxygen reduction reaction. Adv. Funct. Mater. **27**(28), 1700802 (2017). https://doi.org/10.1002/adfm.201700802

[S7] P. Chen, T. Zhou, L. Xing, K. Xu, Y. Tong et al., Atomically dispersed iron-nitrogen species as electrocatalysts for bifunctional oxygen evolution and reduction reactions. Angew. Chem. Int. Ed. **56**(2), 610-614 (2017). https://doi.org/10.1002/ange.201610119

[S8] Q. Li, W. Chen, H. Xiao, Y. Gong, Z. Li et al., Fe isolated single atoms on S, N codoped carbon by copolymer pyrolysis strategy for highly efficient oxygen reduction reaction. Adv. Mater, **30**(25), e1800588 (2018). https://doi.org/10.1002/adma.201800588

[S9] S. Liu, L. Liu, X. Chen, Z. Yang, M. Li et al., On an easy way to prepare Fe, S, N tri-doped mesoporous carbon materials as efficient electrocatalysts for oxygen reduction reaction. Electrocatalysis, **10**(1), 72-81(2018). https://doi.org/10.1007/s12678-018-0496-9

[S10] J. Wang, W. Liu, G. Luo, Z. Li, C. Zhao et al., Synergistic effect of well-defined dual sites boosting the oxygen reduction reaction. Energy Environ. Sci. **11**(12), 3375-3379 (2018). https://doi.org/10.1039/c8ee02656d

[S11] Y. Han, Y. Wang, R. Xu, W. Chen, L. Zheng et al., Electronic structure engineering to boost oxygen reduction activity by controlling the coordination of the central metal. Energy Environ. Sci. **11**(9), 2348-2352 (2018). https://doi.org/10.1039/c8ee01481g

[S12] Y. Chen, S. Ji, S. Zhao, W. Chen, J. Dong et al., Enhanced oxygen reduction with single-atomic-site iron catalysts for a zinc-air battery and hydrogen-air fuel cell. Nat. Commun. **9**(1), 5422 (2018). https://doi.org/10.1038/s41467-018-07850-2

[S13] Y. Zheng, Y. Jiao, Y. Zhu, Q. Cai, A. Vasileff et al., Molecule-level g-C_3N_4 coordinated transition metals as a new class of electrocatalysts for oxygen electrode reactions. J. Am. Chem. Soc. **139**(9), 3336-3339 (2017). https://doi.org/10.1021/jacs.6b13100
